# Antimicrobial consumption in food animals in Fiji: Analysis of the 2017 to 2021 import data

**DOI:** 10.3389/frabi.2022.1055507

**Published:** 2022-12-21

**Authors:** Royford Magiri, Chaminda Dissanayake, Walter Okello

**Affiliations:** ^1^ Fiji National University, College of Agriculture, Fisheries and Forestry, Nausori, Fiji; ^2^ Animal Section, Biosecurity Authority of Fiji (BAF), Suva, Fiji; ^3^ Commonwealth Scientific and Industrial Research Organization, Black Mountain Science and Innovation Park, Acton, ACT, Australia

**Keywords:** antimicrobial resistance, antimicrobial consumption, Fiji, animal biomass, imported antimicrobials, animal

## Abstract

**Introduction:**

Globally, the demand for animal protein for human consumption has beenQ7 Q6increasing at a faster rate in the last 5 to 10 decades resulting in increasedantimicrobial consumption in food producing animals. Antimicrobials arefrequently used as part of modern methods of animal production, which mayput more pressure on evolution of antibiotic resistant bacteria. Despite theserious negative effects on animal and human health that could result fromusing antibiotics, there are no assessment of antimicrobials consumed by thelivestock sector in Fiji as well as other Pacific Island Countries. The objective ofthis study was to quantify antimicrobials imported for consumption in foodanimals into Fiji from 2017 to 2021.

**Methods:**

Data on imported antimicrobials, whichwere finished products, was obtained from Biosecurity Authority Fiji (BAF).Imported antimicrobials were then analyzed by antimicrobial class, andimportance to veterinary and human medicine.

**Results:**

An average of 92.86 kg peryear (sd = 64.12) of antimicrobials as a net weight was imported into Fiji in the2017-2021 study period. The mean amount of imported active antimicrobialingredients after adjusting for animal biomass was 0.86 mg/kg (sd = 0.59). Fromthe total antimicrobial imports during the years 2017 to 2021, penicillins(69.72%) and tetracycline (15.95%) were the most imported antimicrobialclasses. For animal health 96.48% of the antimicrobial imports wereveterinary critically important antimicrobials. For human healthfluroquinolones, macrolides, aminoglycosides, and penicillins were theimported critically important antimicrobials.

**Discussion:**

The study concluded that use ofantimicrobials in food producing animals is low but monitoring of antimicrobialconsumption and antimicrobial resistance was critical in Fiji due to overrelianceon critically important antimicrobials.

## Introduction

Antimicrobial resistance (AMR), which is considered a One Health problem as it occurs between humans, animals, plants, and the ecosystem, has emerged as one of the major global health threats (Prestinaci et al., 2015; Léger et al., 2021). AMR is linked to misuse (i.e., under or overuse) of antimicrobials in humans and animals. Antimicrobials are frequently utilized in food animals to promote growth and prevent and treat animal diseases (Schwarz et al., 2001; McEwen and Fedorka-Cray, 2002; Landers et al., 2012). The prudent use of antimicrobial agents in food producing animals is necessary to prevent the development and spread of antimicrobial resistance between animals and human (Anthony et al., 2001; Lekshmi et al., 2017; Aidara-Kane et al., 2018). However, indiscriminate use of antimicrobials in food producing animals leads to emergence of antimicrobial resistant microorganisms by way of natural selection and can result in decreased benefits gained from antimicrobial effectiveness over time (Cooper and Okello, 2021). Despite this challenge, no previous studies have been conducted on antimicrobial consumption (AMC) in human and animals in Fiji and the pacific. Antimicrobial resistant organisms of animal origin are transmitted to human *via* environment, consumption of animal food products and to animal health worker through direct contact with animals (Economou and Gousia, 2015; Founou et al., 2016; Graham et al., 2019). Human intestines may become colonized with animal-derived, drug-resistant bacteria like *Escherichia coli* and *Enterococcus* species (Phillips et al., 2004; Rousham et al., 2018). People who are frequently exposed, such as those who work in slaughterhouses, food establishments, and farms where animals are fed antibiotics, are more likely to develop resistance to *E. coli* than the general public (Van den Bogaard et al., 2001). There has been a noticeable surge in the appearance of resistant food pathogens such as *Salmonella* spp., *Campylobacter* spp., and other bacteria thought to be markers of AMR as a result of increased usage of antimicrobial drugs in food animals (Sanchez et al., 2002; Elhadidy et al., 2020). Furthermore, repeated exposure to low doses of antimicrobial drugs when used as growth-promoters or for prophylactic treatment in livestock production results in the development of ideal conditions for the emergence and spread of AMR organisms in animals (Chantziaras et al., 2014). To further exacerbate the problem of AMR in developing countries, consumption of antimicrobials in animals is set to increase exponentially over the coming decades particularly in low and middle income countries (Klein et al., 2018; Van Boeckel et al., 2019). Increased AMC in low and middle income countries is partly due to rising incomes resulting in increased demand for animal protein which necessitate the use of antimicrobials to increase livestock productivity (Rushton, 2015; Kirchhelle, 2018; Manyi-Loh et al., 2018).

The unprecedented increase in AMR has led to the development of a global strategy which includes monitoring of AMC in animals (Schar et al., 2018; Munkholm and Rubin, 2020). Monitoring of AMC enables detection of risk factors as well as understanding temporal association between AMC and AMR (Page and Gautier, 2012). Such analysis provides evidence for the development of policies for managing AMR both in human and animal health (Ferreira, 2017). Furthermore, some of the antimicrobials used in food producing animals are also used in humans to treat common infections hence development of resistance in animal has a great economic impact on human health (Magouras et al., 2017). At the global level, the World Organization for Animal Health (WOAH), founded as the Office International des Epizooties (OIE), has documented harmonized guidelines for AMC monitoring which includes sources of AMC data such as import data, sales, manufacturing, and farm use data (World Organisation for Animal Health, 2020a). Additionally, WOAH and the World Health Organization (WHO) have documented antimicrobial agents of veterinary and human health importance respectively (World Health Organization, 2019; World Organisation for Animal Health, 2021). Although, some countries have been collecting data on AMC, this has mostly been done in developed countries (Grave et al., 2010; Hosoi et al., 2013; Hillerton et al., 2017). Low and middle income countries face numerous challenges such as lack of data on antimicrobial use (AMU) mostly due to limited veterinary services (Tiseo et al., 2020).

Fiji is one of the Pacific Island countries in the Oceania region with the majority of the population depending on subsistence agriculture and keeps several livestock species such as cattle, chicken, sheep, and goat. The country has three hundred islands, but majority of the population lives in two main islands namely Viti Levu and Vanua Levu. Livestock keeping in Fiji is important as it is a source of income, protein, and weed control. According to the 2020 agricultural census, there were 119,691 cattle, 37,435 sheep, 143,853 goats, and 1,412, 901 chicken (Ministry of Agriculture, 2020). Despite the importance of livestock, there has been limited studies on animal diseases with brucellosis, and bovine tuberculosis being the most studied (Tukana et al., 2016; Borja et al., 2018). Additionally, the prevalence of AMR in food animals in Fiji remain unknown (Magiri et al., 2022). Lack of information on animal health issues in Fiji could be limited due to limited veterinary services; animal health providers have also been found to have limited knowledge on AMR (Khan et al., 2022a; Khan et al., 2022b).

The aim of this study is to address the gaps in understanding AMC in food animals in Fiji at the national level using antimicrobials imported between 2017 and 2021. The imported antimicrobials are described according to their antimicrobial class and their importance in veterinary and human medicine. The findings can be useful for risk analysis and planning, evaluation of cost-effectiveness of initiatives to promote prudent antimicrobial usage, and development of strategies to reduce AMR.

## Materials and methods

### Data collection and characterization of imported antimicrobials

The data on imported antimicrobials between 2017 and 2021 was obtained, after seeking approval, directly from the official records of the Biosecurity Authority of Fiji (BAF). The BAF is a Public Enterprise under the Public Enterprises Act 2019 tasked with managing quarantine control at the Fiji border and provision of import and export inspection and certification. The Database of the imported antimicrobials for veterinary use contained name of importer, date of importation, active ingredients imported as finished products, package sizes, and antimicrobial chemical compound, and represents a tier 1 distribution system. All veterinary drugs imported into Fiji including antimicrobials have to be registered by BAF. Only the veterinary antimicrobials import data was obtained from BAF. The data was screened for quantity imported, recommendation for use in food animals, name of active ingredient, and concentration of active ingredient. Characterization of the extracted data was done using OIE list of antimicrobials of veterinary importance and the WHO list of antimicrobials of human health importance (World Health Organization, 2019; World Organisation for Animal Health, 2021). Also, the data was stored in Microsoft Excel (Microsoft Corporation, Redmond, WA, USA).

### Animal biomass estimation

Animal biomass, which was the total number of food animals in Fiji in tons, was estimated from animal population, animal slaughter, quantity of meat produced, carcass weight, and live animal weight data with cattle, sheep, goats, pigs, and chicken being the major focus as they are the most consumed in Fiji. Regarding livestock population, the 2009 and 2020 agricultural census (Ministry of Agriculture, 2009; Ministry of Agriculture, 2020) was first used to estimate the annual population growth rate using the equation below.


(1) $$r\;={\left(P_{2}/P_{1}\right)}^{\frac{1}{y}}\;-\;1$$

Where r is annual growth rate of a particular livestock species, P_2_ is the present livestock population (i.e., 2020) for a particular livestock species (e.g., cattle, sheep, goat, pigs, or chicken), P_1_ is the past livestock population (i.e., 2009) livestock population for a particular livestock species, y is the number of years between the present and past years which was 11 years in this case. Number of animals slaughtered, and quantity of meat produced was obtained from Fiji meat industry report (Fiji meat industry board, 2016). However, number of chickens slaughtered, and quantity produced was obtained from FAOSTAT as this information was not available in the Fiji livestock industry report (Food and Agriculture Organization of the United Nations). Carcass weight was estimated by dividing total weight of animals slaughtered with total number of animals slaughtered whereas live weight was estimated by dividing carcass weight by conversion coefficient for a particular livestock species; cattle, sheep, goat, pig, and chicken conversion factors used in this study were 0.7, 0.47, 0.47, 0.78, and 0.7 respectively (Eurostat, 2009). The total animal biomass from 2017 to 2021 was calculated as described by the OIE (Góchez et al., 2019) except for cattle which was calculated by multiplying live weight with the cattle population due to lack of data on proportion of livestock slaughtered and quantity of meat for different age groups. More information on the animal biomass calculations can be found in the **Supplementary Material**.

### Data analysis

To obtain the quantity of imported antimicrobials, the amount of each antimicrobial agent (chemical compound as declared in import permit) per package was calculated first, and the result subsequently multiplied by the number of packages imported to obtain the overall amount of antimicrobial agent, which was converted to kilograms as per the OIE recommendation (World Organisation for Animal Health, 2020b). Equation 2 was used to calculate the total amount (first as milligram then converted to grams) of antimicrobial agent in a container (e.g., bottles and syringes).


(2) $$Total\;amount\;of\;antimicrobial\;agent\;in\;a\;container\;\left(g\right)=\left(strength\;\left(\frac{mg}{ml}\right)\times container\;size\;\left(ml\right)\right)/1000$$

Where mg is milligram and ml is milliliter.

Afterwards, the content of the antimicrobial agent per package was calculated using Equation 3.


(3) $$\begin{array}{c} Content\;of\;antimicrobial\;agent\;per\;package\;(g)\\ =Total\;amount\;of\;antimicrobial\;agent\;in\;a\;container\\ \;(g)\;x\;number\;of\;packs \end{array}$$

The number of packs were 4, 6, 10, 12, and 20. However, some importers occasionally imported single units.

Equation 4 was used to calculate the total amount of antimicrobial agent in a blister or a strip.


(4) $$\begin{array}{c} Content\;of\;antimicrobial\;agent\;per\;blister\;pack\;(g)\\ =(strength\;per\;tablet\;(mg)\;x\;number\;of\;blisters\\ \;x\;number\;of\;tablets\;in\;in\;each\;blister)/1000 \end{array}$$

For antimicrobial agents that were reported using international units (UI) such as penicillin for intramuscular injection, conversion factors were used to convert this into mg/ml (World Organisation for Animal Health, 2020b). Equations 2 and 3 were then used to derive the content of antimicrobial agent per package. All weights of the imported active antimicrobial ingredients were expressed in kilograms except when adjusting for animal biomass which was done in milligrams. **Tables S1, S2** in the Supplementary Materials show how the antimicrobial quantities for each antimicrobial agent was derived. The antimicrobials were mostly imported from Australia, New Zealand, India, and United Kingdom.

Antimicrobials used in food animals was adjusted for the relevant animal biomass by dividing antimicrobial agents imported in milligrams (mg) by the total animal biomass in kg (Góchez et al., 2019). The standard weight for sheep and goats used in this study for calculating their biomass was 37.5 kilograms (Galal, 2005). Trend analysis was done using Mann Kendall test in R Software (package = Kendall) to determine whether time series of the imported antimicrobials had an upward or downward monotonic trend (McLeod, 2022). However, the trend analysis is not the best form of presenting a data of a very short period. The hypothesis was that there was a trend in the imported antimicrobials. Apart from determining quantities of antimicrobials imported and their trend, antimicrobials of both veterinary and human importance were quantified between 2017 and 2021. Data analysis was done using R Software (R Core Team, 2022).

## Results

**Figure 1:**
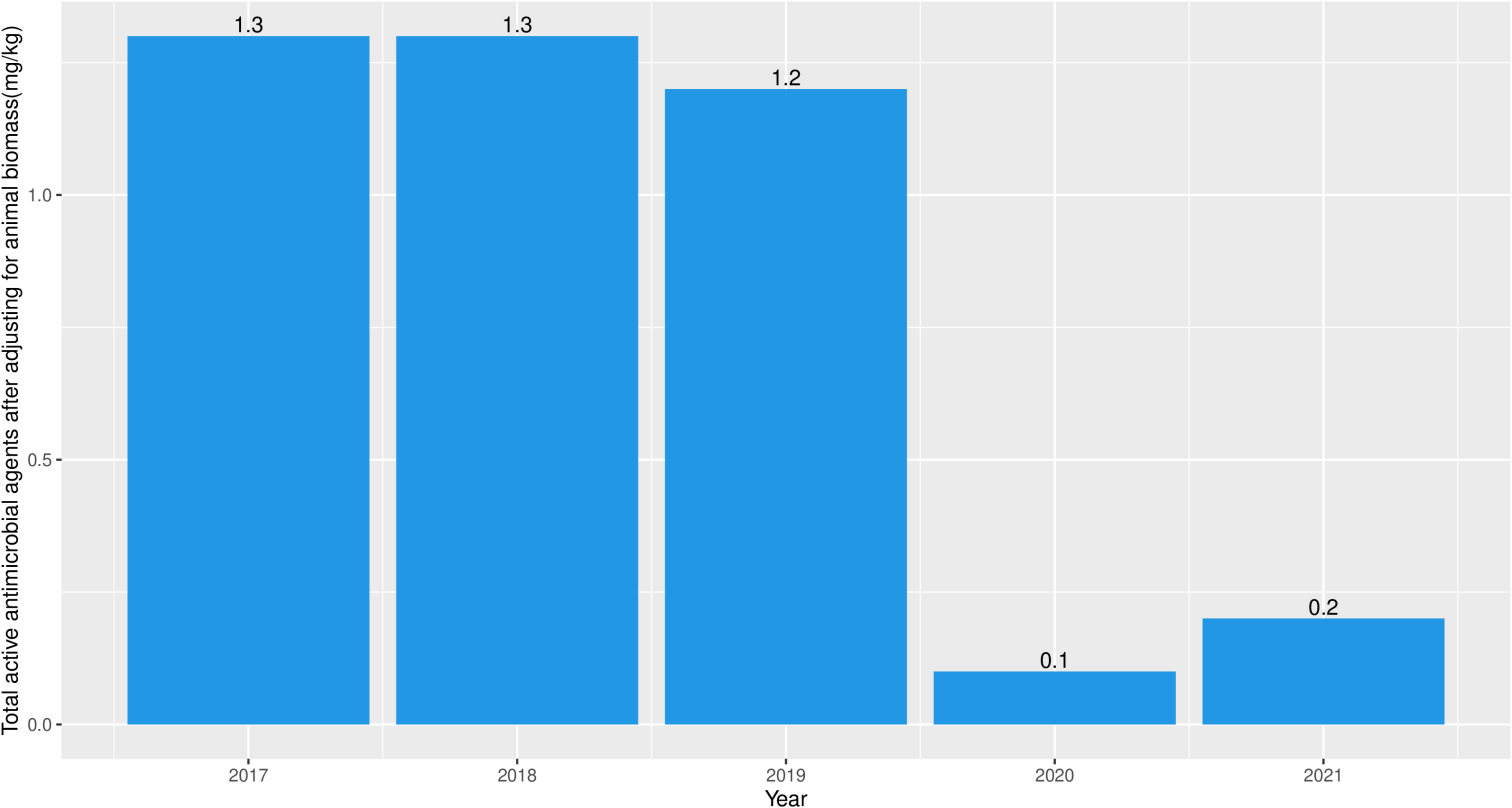
Antimicrobial import weight (mg) adjusted by animal biomass (kg) into Fiji between 2017 and 2021.

A total of 464.31 kg of active antimicrobial agents (**Table 1**), which were all finished products, was imported to Fiji between 2017 and 2021 for use in food animals (mean = 92.86 kg per year, standard deviation (sd) = 64.12 kg per year). Notably, all antimicrobials for use in animals, were recorded by BAF at the point of entry. The annual quantities and antimicrobial classes imported over the study period is as shown in **Table 1**. We assumed that no antimicrobial that entered the country through Illegal route of importation which is usually a major problem in developing countries. The mean amount of imported antimicrobials after adjusting for animal biomass was 0.86 mg/kg (sd = 0.59). Additionally, the mean amount of imported antimicrobials after adjusting for animal biomass in 2017, 2018, 2019, and 2021 was 1.3 mg/kg, 1.3 mg/kg, 1.2 mg/kg, and 0.2 mg/kg respectively (**Figure 1**). The antimicrobial chemical compound names of the imported finished products included gentamycin sulphate, cephalothin sodium, cephazolin sodium, cefuroxime sodium, ciprofloxacin hydrochloride, norfloxacin, lincomycin hydrochloride monohydrate, erythromycin, penicillin G procaine, silver sulfadiazine, sulfamethoxazole, tetracycline hydrochloride, and metronidazole. Trend analysis revealed that there was no significant increasing or decreasing trend in the antimicrobials imported between 2017 and 2021 (test statistic: -0.20; p-value: 0.80).

**Table 1:** Annual quantities of antimicrobials imported in Fiji between 2017 and 2021.

Imported antimicrobial agents (class, sub-class)	Annual quantities of imported active antimicrobial agents (kg, %)					
	2017	2018	2019	2020	2021	Total
Aminoglycosides	0.29 (0.22)	0 (0)	0.16 (0.10)	0.14 (0.73)	1.39 (5.05)	1.98 (0.43)
Cephalosporins						
First-generation cephalosporin	0.83 (0.64)	0.1 (0.01)	0.43 (0.28)	0.08 (0.42)	0.60 (2.18)	1.94 (0.42)
Second-generation cephalosporin	0 (0)	0 (0)	0.01 (0.01)	0 (0)	0 (0)	0 (0)
Quinolones						
Fluoroquinolone	0.01 (0.01)	0.01 (0.01)	0.1 (0.07)	0 (0)	0.01 (0.04)	0.13 (0.03)
Lincosamides	14.25 (10.97)	0 (0)	0.04 (0.03)	0.03 (0.16)	0.05 (0.18)	14.36 (3.09)
Macrolides	0.02 (0.02)	28.75 (21.44)	0.01 (0.01)	0 (0)	0 (0)	28.78 (6.20)
Penicillins	103.16 (79.40)	101.84 (75.95)	94.33 (61.43)	13.63 (70.92)	10.78 (39.19)	323.73 (69.72)
Sulfonamides	1.85 (1.42)	0 (0)	1.35 (0.88)	3.29 (17.12)	12.57 (45.69)	19.06 (4.11)
Tetracyclines	9.49 (7.30)	3.48 (2.60)	57.06 (37.16)	2.06 (10.72)	1.97 (7.16)	74.06 (15.95)
Nitroimidazoles	0.03 (0.02)	0 (0)	0.08 (0.05)	0 (0)	0.15 (0.55)	0.26 (0.06)
Total	129.93 (27.98)	134.08 (28.87)	153.56 (33.07)	19.22 (4.13)	27.51 (5.92)	464.31 (100)

A total of 13 antimicrobial active ingredients (namely gentamycin, cephalothin, cephazolin, cefuroxime, ciprofloxacin, norfloxacin, lincomycin, erythromycin, penicillin, sulfadiazine, sulfamethoxazole, tetracycline, and metronidazole belonging to nine antimicrobial classes (namely aminoglycosides, cephalosporins, quinolones, lincosamides, macrolides, penicillins, sulfonamides, tetracycline, and nitroimidazoles) was reported. Also, screening of the antimicrobial agents imported revealed that no nitrofuran was imported during the study period. Analysis of the imported antimicrobial agents between 2017 and 2021 revealed that 69.72% of the total imported antimicrobials within the study period were penicillins (**Table 1**). Another commonly imported antimicrobials were tetracyclines (15.95%); penicillins and tetracyclines comprised 85.64% of the total imported antimicrobials between 2017 and 2021.

Analysis of the imported antimicrobial agents according to animal health importance revealed that penicillins (72.30%) were the top veterinary critically important antimicrobials during the years 2017 to 2021 followed by tetracyclines (16.54%) (**Table 2**). Critically important antimicrobial agents in animal health are the limited agents available to treat serious infections in animals. The definition of clinically important antimicrobials is similar in animal health, but the serious infections include those from non-human sources. In human health, penicillins are regarded as critically important, high priority antimicrobials. Tetracycline was the second most imported veterinary critically important antimicrobial (16.54%) (**Table 2**). However, in human health, tetracycline is not regarded as a critically important antimicrobial. Other antimicrobial agents of both veterinary and medical critical importance imported in Fiji between 2017 and 2021 included fluoroquinolones and macrolides (**Table 2**). Results for highly important antimicrobial for animal use, revealed that linconsamides were the most imported (**Table 2**). No veterinary important antimicrobial was imported during the studied period.

**Table 2:** Total quantities (in kg) of antimicrobials imported in Fiji (2017-2021) according to animal and human health importance.

Imported antimicrobial agents (class, sub-class)	Total imported active antimicrobial agents according to veterinary importance (kg, %)		
	Veterinary critically important	Veterinary highly important	Veterinary important
Aminoglycosides^2^	1.98(0.44)	–	–
Cephalosporins			
First-generation cephalosporin	–	1.94(11.89)	
Second-generation cephalosporin	–	0.01(0.06)	
Quinolones			
Fluoroquinolones^1^	0.13(0.02)	–	
Lincosamides	–	14.36(88.05)	
Macrolides^1^	28.78(6.43)	–	–
Penicillins^2^	323.73(72.30)	–	–
Sulfonamides	19.06(4.26)	–	–
Tetracyclines	74.06(16.54)	–	–
**Total**	447.73(96.48)	16.31(3.52)	–

^1^Critically important, highest priority antimicrobial agent in human health.

^2^Critically important, high priority antimicrobial agent in human health.

## Discussion

To the best of our knowledge, this is the first study in Fiji and within the broader Pacific Island countries to describe imported antimicrobial agents for food animals using international guidelines. Fiji imports all antimicrobials therefore this study was an important proxy for understanding AMC in animal health at the national level; obtaining data on AMU at the farm level or retail is challenging due to lack of records. The study also forms a baseline for analyzing future trends in AMC in food animals in Fiji and the Pacific.

The quantity of antimicrobials imported for use in food animals, adjusted for animal biomass, in Fiji was found to be 0.86 mg/kg on average compared to an average consumption of 237.72mg/kg in Oceania, Asia, and Far East, and a global average of 144.39 mg/kg antimicrobials in livestock (World Organisation for Animal Health, 2020b). Equally, in New Zealand, which is one of the countries in Oceania, AMC in food animals was found to be 9.4mg active ingredient/kg biomass (Hillerton et al., 2017). In Pakistan, AMC was found to be 10.05 mg/kg of the cumulative animal biomass, while in sub-Saharan Africa, it was found to be 5.24 ± 1.40 mg/population correction unit (Mouiche et al., 2020; Umair et al., 2022). Studies in Timor-Leste, which is a low and middle income country with a relatively similar agricultural system like Fiji, AMC in food animals was reported to be 0.55 mg/kg after adjusting for animal biomass (Ting et al., 2021).

The low consumption of antimicrobials in Fiji could be due to several factors such as low livestock population, relatively low occurrence of animal diseases, and less intensified livestock production systems. However, further studies are required in Fiji to determine the prevalence of animal diseases including farming practices especially AMU. A past study showed that farmers knowledge of AMR in Fiji is low (Khan et al., 2021; Khan et al., 2022a; Khan et al., 2022b). Another important observation on the quantities of imported antimicrobial agents, was the sharp decrease of imported antimicrobials in 2020 and 2021. The COVID-19 pandemic could be responsible for this decrease as Fiji relies on imported antimicrobials. This also shows how vulnerable Pacific Island Countries are to external shocks such as pandemics which may affect food security (Singh et al., 2022).

Analysis of the imported antimicrobial agents according to their importance in veterinary and human medicine, revealed that most antimicrobials imported for consumption in food animals are considered to be veterinary critically important; of the total antimicrobials imported for veterinary use between 2017 and 2021, 96.48% were veterinary critically important. This requires Fiji to judiciously use antimicrobials for food production to prevent a high risk of AMR occurrence which would render the antimicrobials ineffective and ultimately resulting in food insecurity. Furthermore, this study found that penicillins and tetracyclines are the most commonly imported antibiotics indicating overreliance on broad-spectrum antibiotics for treatment. Penicillins and tetracyclines are commonly used by farmers in developing countries due to their low cost and broad-spectrum antimicrobial activity (Beyene et al., 2015). Importation of fluroquinolone, which pose higher risk to public health regarding, and macrolides and penicillins, both of which pose limited risk to public health, need to be monitored in Fiji to prevent AMR occurrence in humans in Fiji. Monitoring for AMR is therefore a recommendation based on the study findings. A positive finding was that nitrofuran was not imported into Fiji during the study period. Several toxicological studies have revealed that nitrofuran drugs may have carcinogenic properties posing a major public health risk; use of nitrofurans in food animals has been banned by the European Union (McCalla, 1979; Antunes et al., 2006).

The study had limitations and challenges. First, data on AMC in the livestock sector in Fiji and the broader Pacific Island Countries is limited due to both the lack of comprehensive government level surveillance systems resulting from shortage of veterinarians and the reluctance of livestock industry (food animal producers and animal feed producers) to give the comprehensive reports on antimicrobial consumption. In this study, data was from imported antimicrobials which represent a tier 1 distribution system. Imported antimicrobials data (tier 1 systems) may over or underestimate the actual quantities of antimicrobials consumed compared to data obtained from either retailers, veterinarians, or producers. However, farmers, veterinarians, retailers, and producers do not regularly keep data on AMU due to insufficient enforcement by regulatory authorities in Fiji (Magiri et al., 2022). Therefore, this study assumed that data on imported antimicrobials can be the best proxy for ascertaining quantities of antimicrobials consumed by food animals in Fiji nationally. Second, there was difficulty in obtaining parameters for estimating animal biomass (e.g., annual livestock population, number of livestock slaughtered, quantities of meat etc.). Livestock census in Fiji is done every ten years but the actual number of livestock per year is usually unavailable. This study mostly relied on country available data rather than FAOSTAT as these were deemed to be more reliable; FAOSTAT uses imputation methods to estimate number of livestock slaughtered and quantities of meat harvested. Additionally, the OIE estimation of AMC globally, relies on European parameters (e.g., standard weights) which could slightly overestimate animal biomass. Parameters that closely represented Fiji agricultural production systems was used in this study to enable accurate estimation of the animal biomass.

In conclusion, this study found that AMC in food animals is relatively low in Fiji possibly due to the subsistence nature of livestock production and low livestock population. However, overreliance on antimicrobials of last resort for livestock production as well as importation of antimicrobials of critical importance to human health warrant regular monitoring of AMU and AMR in Fiji for food security and protection of public health. The current Australia Centre for International Agricultural Research (ACIAR) funded AMR project is aimed at addressing some of the gaps in managing AMR in the region. The project is the first to adopt the One-Health approach to research into AMR in humans, animals and the environment in the Pacific region.

## Data availability statement

The original contributions presented in the study are included in the article/**Supplementary Material**. Further inquiries can be directed to the corresponding author.

## Ethics statement

The animal study was reviewed and approved by CSIRO Health and Medical Human Research Ethics Committee (CHMHREC) approval 2020_113_RR as well as Fiji Human Health Research and Ethics Review Committee (FNHRERC number 25/2020).

## Author contributions

Equal contribution: RM, CD, and WO contributed equally to this work. RM: Conceived the idea, analyzed the data, and edited manuscript. CD: Collected and analyzed the data. WO: Analyzed the data, edited the manuscript and provided resources for publication. All authors contributed to the article and approved the submitted version.
