# Supplementary material for: Antimicrobial consumption in food animals in Fiji: Analysis of the 2017 to 2021 import data
Source: Front Antibiot. 2022 Dec 21;1:1055507. doi: 10.3389/frabi.2022.1055507 (PMC11736331; doi:10.3389/frabi.2022.1055507)
Supplement: Supplementary file 1 [file Table_1.docx]

Supplementary Material

**Table S1: Imported injectable antimicrobial agents in Fiji between 2017 and 2021**

| Year | Antimicrobial agent | Strength (mg/ml) | Container size (ml) | Total amount of active ingredient per container (mg) | Total amount of active ingredient per container (g) | Package size | Number of packages | Total amount of active ingredient in the packages (g) | Total amount of active ingredient in the packages (kg) |
| --- | --- | --- | --- | --- | --- | --- | --- | --- | --- |
| 2017 | Gentamycin | 50 | 100 | 5000 | 5 | 12 | 4 | 240 | 0.24 |
| 2017 | Gentamycin | 50 | 50 | 2500 | 2.5 | 10 | 2 | 50 | 0.05 |
| 2018 | Gentamycin | 0 | 0 | 0 | 0 | 0 | 0 | 0 | 0 |
| 2019 | Gentamycin | 100 | 100 | 10000 | 10 | 4 | 4 | 160 | 0.16 |
| 2020 | Gentamycin | 50 | 100 | 5000 | 5 | 4 | 6 | 120 | 0.12 |
| 2020 | Gentamycin | 100 | 100 | 10000 | 10 | 4 | 1 | 40 | 0.04 |
| 2021 | Gentamycin | 50 | 100 | 5000 | 5 | 12 | 23 | 1380 | 1.38 |
| 2021 | Gentamycin | 100 | 100 | 10000 | 10 | 1 | 1 | 10 | 0.01 |
| 2017 | Cephalothin | 10 | 10 | 100 | 0.1 | 12 | 692 | 830.4 | 0.83 |
| 2018 | Cephalothin | 10 | 10 | 100 | 0.1 | 12 | 10 | 12 | 0.01 |
| 2019 | Cephalothin | 10 | 10 | 100 | 0.1 | 12 | 350 | 420 | 0.42 |
| 2019 | Cefazolin | 10 | 10 | 100 | 0.1 | 12 | 10 | 12 | 0.01 |
| 2020 | Cephalothin | 10 | 10 | 100 | 0.1 | 12 | 65 | 78 | 0.08 |
| 2021 | Cephalothin | 10 | 10 | 100 | 0.1 | 12 | 500 | 600 | 0.60 |
| 2017 | Cefuroxime | 0 | 0 | 0 | 0 | 0 | 0 | 0 | 0.00 |
| 2018 | Cefuroxime | 0 | 0 | 0 | 0 | 0 | 0 | 0 | 0.00 |
| 2019 | Cefuroxime | 25 | 10 | 250 | 0.25 | 20 | 2 | 10 | 0.01 |
| 2020 | Cefuroxime | 0 | 0 | 0 | 0 | 0 | 0 | 0 | 0.00 |
| 2021 | Cefuroxime | 0 | 0 | 0 | 0 | 0 | 0 | 0 | 0.00 |
| 2017 | Ciprofloxacin | 100 | 50 | 5000 | 5 | 1 | 2 | 10 | 0.01 |
| 2018 | Norfloxacin | 100 | 50 | 5000 | 5 | 1 | 2 | 10 | 0.01 |
| 2019 | Ciprofloxacin | 100 | 50 | 5000 | 5 | 1 | 20 | 100 | 0.10 |
| 2020 | Ciprofloxacin/Norfloxacin | 0 | 0 | 0 | 0 | 0 | 0 | 0 | 0.00 |
| 2021 | Ciprofloxacin | 100 | 50 | 5000 | 5 | 1 | 2 | 10 | 0.01 |
| 2017 | Lincosamide | 200 | 50 | 10000 | 10 | 12 | 117 | 14040 | 14.04 |
| 2017 | Lincosamide | 100 | 100 | 10000 | 10 | 10 | 2 | 200 | 0.20 |
| 2017 | Lincosamide | 100 | 30 | 3000 | 3 | 2 | 1 | 6 | 0.01 |
| 2018 | Lincosamide | 0 | 0 | 0 | 0 | 0 | 0 | 0 | 0.00 |
| 2019 | Lincosamide | 100 | 50 | 5000 | 5 | 1 | 2 | 10 | 0.01 |
| 2020 | Lincosamide | 100 | 50 | 5000 | 5 | 1 | 7 | 35 | 0.04 |
| 2021 | Lincosamide | 100 | 100 | 10000 | 10 | 1 | 5 | 50 | 0.05 |
| 2017 | Erythromycin | 200 | 100 | 20000 | 20 | 1 | 1 | 20 | 0.02 |
| 2018 | Erythromycin | 200 | 100 | 20000 | 20 | 12 | 117 | 28080 | 28.08 |
| 2018 | Erythromycin | 200 | 50 | 10000 | 10 | 12 | 5 | 600 | 0.60 |
| 2018 | Erythromycin | 200 | 30 | 6000 | 6 | 6 | 2 | 72 | 0.07 |
| 2019 | Erythromycin | 200 | 50 | 10000 | 10 | 1 | 1 | 10 | 0.01 |
| 2020 | Erythromycin | 0 | 0 | 0 | 0 | 0 | 0 | 0 | 0.00 |
| 2021 | Erythromycin | 0 | 0 | 0 | 0 | 0 | 0 | 0 | 0.00 |
| 2017 | Penicillin | 100 | 10 | 1000 | 1 | 20 | 30062 | 601240 | 601.24 |
|  | Penicillin | 1800 | 100 | 180000 | 180 | 12 | 100 | 216000 | 216.00 |
|  | Penicillin | 1800 | 50 | 90000 | 90 | 12 | 11 | 11880 | 11.88 |
|  | Penicillin | 1800 | 250 | 450000 | 450 | 1 | 1 | 450 | 0.45 |
| 2018 | Penicillin | 100 | 10 | 1000 | 1 | 20 | 599 | 11980 | 11.98 |
|  | Penicillin | 100 | 10 | 1000 | 1 | 1 | 5 | 5 | 0.01 |
| 2019 | Penicillin | 100 | 10 | 1000 | 1 | 20 | 15556 | 311120 | 311.12 |
|  | Penicillin | 1800 | 100 | 180000 | 180 | 12 | 47 | 101520 | 101.52 |
|  | Penicillin | 1800 | 50 | 90000 | 90 | 12 | 6 | 6480 | 6.48 |
|  | Penicillin | 1800 | 250 | 450000 | 450 | 1 | 1 | 450 | 0.45 |
|  | Penicillin | 100 | 10 | 1000 | 1 | 1 | 5 | 5 | 0.01 |
| 2020 | Penicillin | 1800 | 50 | 90000 | 90 | 12 | 12 | 12960 | 12.96 |
|  | Penicillin | 100 | 10 | 1000 | 1 | 20 | 33 | 660 | 0.66 |
|  | Penicillin | 100 | 10 | 1000 | 1 | 1 | 5 | 5 | 0.01 |
| 2021 | Penicillin | 100 | 10 | 1000 | 1 | 20 | 498 | 9960 | 9.96 |
|  | Penicillin | 1800 | 50 | 90000 | 90 | 1 | 9 | 810 | 0.81 |
|  | Penicillin | 100 | 10 | 1000 | 1 | 1 | 5 | 5 | 0.01 |
| 2017 | Sulfadiazine | 200 | 50 | 10000 | 10 | 12 | 14 | 1680 | 1.68 |
|  | Sulfadiazine | 200 | 100 | 20000 | 20 | 1 | 8 | 160 | 0.16 |
|  | Sulfadiazine | 200 | 50 | 10000 | 10 | 1 | 1 | 10 | 0.01 |
| 2018 | Sulfadiazine | 0 | 0 | 0 | 0 | 0 | 0 | 0 | 0 |
| 2019 | Sulfadiazine | 200 | 50 | 10000 | 10 | 12 | 7 | 840 | 0.84 |
|  | Sulfamethoxazole | 200 | 50 | 10000 | 10 | 12 | 4 | 480 | 0.48 |
|  | Sulfadiazine | 200 | 50 | 10000 | 10 | 1 | 3 | 30 | 0.03 |
| 2020 | Sulfadiazine | 200 | 50 | 10000 | 10 | 12 | 27 | 3240 | 3.24 |
|  | Sulfamethoxazole | 200 | 50 | 10000 | 10 | 1 | 5 | 50 | 0.05 |
| 2021 | Sulfadiazine | 200 | 50 | 10000 | 10 | 12 | 51 | 6120 | 6.12 |
|  | Sulfamethoxazole | 200 | 50 | 10000 | 10 | 12 | 53 | 6360 | 6.36 |
|  | Sulfadiazine | 200 | 50 | 10000 | 10 | 1 | 9 | 90 | 0.09 |
| 2017 | Tetracycline | 100 | 500 | 50000 | 50 | 12 | 13 | 7800 | 7.8 |
|  | Tetracycline | 100 | 100 | 10000 | 10 | 12 | 13 | 1560 | 1.56 |
|  | Tetracycline | 100 | 50 | 5000 | 5 | 1 | 25 | 125 | 0.13 |
| 2018 | Tetracycline | 100 | 500 | 50000 | 50 | 12 | 4 | 2400 | 2.4 |
|  | Tetracycline | 100 | 100 | 10000 | 10 | 12 | 8 | 960 | 0.96 |
|  | Tetracycline | 100 | 50 | 5000 | 5 | 1 | 24 | 120 | 0.12 |
| 2019 | Tetracycline | 100 | 500 | 50000 | 50 | 12 | 70 | 42000 | 42 |
|  | Tetracycline | 100 | 100 | 10000 | 10 | 12 | 98 | 11760 | 11.76 |
|  | Tetracycline | 100 | 50 | 5000 | 5 | 1 | 259 | 1295 | 1.30 |
| 2020 | Tetracycline | 100 | 500 | 50000 | 50 | 12 | 3 | 1800 | 1.8 |
|  | Tetracycline | 100 | 100 | 10000 | 10 | 12 | 2 | 240 | 0.24 |
|  | Tetracycline | 100 | 50 | 5000 | 5 | 1 | 4 | 20 | 0.02 |
| 2021 | Tetracycline | 100 | 500 | 50000 | 50 | 12 | 3 | 1800 | 1.8 |
|  | Tetracycline | 100 | 50 | 5000 | 5 | 1 | 34 | 170 | 0.17 |
| 2017 | Metronidazole | 100 | 50 | 5000 | 5 | 6 | 1 | 30 | 0.03 |
| 2018 | Metronidazole | 0 | 0 | 0 | 0 | 0 | 0 | 0 | 0 |
| 2020 | Metronidazole | 0 | 0 | 0 | 0 | 0 | 0 | 0 | 0 |
| 2021 | Metronidazole | 100 | 50 | 5000 | 5 | 6 | 5 | 150 | 0.15 |

**Table S2: Imported oral antimicrobial agents in Fiji between 2017 and 2021**

| Year | Antimicrobial agent | Strength (mg per tablet) | Number of blisters per box | Number of tablets per blister per box | Total active ingredient per box (mg) | Total active ingredient per box (g) | Total number of boxes imported | Total active ingredients in all imported boxes (kg) |
| --- | --- | --- | --- | --- | --- | --- | --- | --- |
| 2019 | Tetracycline | 2000 | 20 | 5 | 200000 | 200 | 10 | 2 |
| 2019 | Metronidazole | 1000 | 10 | 4 | 40000 | 40 | 2 | 0.08 |

**Calculation of biomass**

Biomass of different animal species was calculated using the following equations (Eq S1-4)

$Bovine biomass=Cattle population \times Liveweight$ EQ.S1

Cattle population was obtained from the Fiji National Livestock Census (2009 and 2020). Liveweight was estimated by dividing the carcass weight with the cattle conversion factor of 0.7 (Ting et al 2021). The carcass weight was obtained by the quantity of meat harvested by the number of animals slaughtered. Information on quantities of animals slaughtered and meat harvested was obtained from the Fiji Meat Industry report (2017).

$$Goat and sheep biomass=\left( Animals slaughtered \times Live weight \right)$$

$+[Goat or Sheep population-(\frac{Animals slaughtered}{1.5})\times37.5kg]$ EQ.S2

Goat and sheep liveweight was estimated by dividing the carcass weight with a conversion factor of 0.47 (Ting et al 2021)

$Chicken biomass=animals slaughtered\times Live weight$ EQ.S3

$Pig biomass=\left( Animals slaughtered \times Live weight \right)+(Pig population \times sow weight \times0.09)$ EQ.S4

Pig liveweight was estimated by dividing the carcass weight with a conversion factor of 0.47 (Ting et al 2021). The sow weight used was 240kg (Gochez et al 2019).

**Table S3: Animal biomass for cattle, chicken, goats, pigs and sheep in Fiji between 2017 and 2021**

| Species | Year | Animal population | Animals slaughtered | Meat harvested (tonnes) | Carcass weight (kg) | Liveweight (kg) | Animal biomass (tonnes) | Data sources |
| --- | --- | --- | --- | --- | --- | --- | --- | --- |
| Cattle | 2015 | 126170.7 | 8471.0 | 2042.0 | 241.1 | 344.4 | 43449.2 | Fiji agricultural census 2009 and 2020; Fiji Meat Industry report 2017 |
|  | 2016 | 124847.3 | 8327.0 | 2240.0 | 269.0 | 384.3 | 47977.8 | Fiji agricultural census 2009 and 2020; Fiji Meat Industry report 2017 |
|  | 2017 | 123537.7 | 8381.0 | 2068.0 | 246.7 | 352.5 | 43546.8 | Fiji agricultural census 2009 and 2020; Fiji Meat Industry report 2017 |
|  | 2018 | 122242.0 | 8293.1 | 2046.3 | 246.7 | 352.5 | 43090.0 | Fiji agricultural census 2009 and 2020; Fiji Meat Industry report 2017 |
|  | 2019 | 120959.8 | 8206.1 | 2024.8 | 246.7 | 352.5 | 42638.1 | Fiji agricultural census 2009 and 2020; Fiji Meat Industry report 2017 |
|  | 2020 | 119691.0 | 8120.0 | 2003.6 | 246.7 | 352.5 | 42190.8 | Fiji agricultural census 2009 and 2020; Fiji Meat Industry report 2017 |
|  | 2021 | 118435.6 | 8034.9 | 1982.6 | 246.7 | 352.5 | 41748.3 | Fiji agricultural census 2009 and 2020; Fiji Meat Industry report 2017 |
| Chicken | 2015 | 2179993.2 | 11183.0 | 23400.0 | 2.1 | 3.0 | 33428.6 | Fiji agricultural census 2009 and 2020 for chicken population; FAOSTAT for animals slaughtered and meat harvested |
|  | 2016 | 1998878.8 | 11398.0 | 15115 | 1.3 | 1.9 | 21592.9 | Fiji agricultural census 2009 and 2020 for chicken population; FAOSTAT for animals slaughtered and meat harvested |
|  | 2017 | 1832811.4 | 11755.0 | 33071 | 2.8 | 4.0 | 47244.3 | Fiji agricultural census 2009 and 2020 for chicken population; FAOSTAT for animals slaughtered and meat harvested |
|  | 2018 | 1680540.9 | 11971.0 | 35499 | 3.0 | 4.2 | 50712.9 | Fiji agricultural census 2009 and 2020 for chicken population; FAOSTAT for animals slaughtered and meat harvested |
|  | 2019 | 1540921.1 | 12256.0 | 34188 | 2.8 | 1.9 | 72740.4 | Fiji agricultural census 2009 and 2020 for chicken population; FAOSTAT for animals slaughtered and meat harvested |
|  | 2020 | 1412901.0 | 12256.0 | 25368 | 2.1 | 4.4 | 53974.5 | Fiji agricultural census 2009 and 2020 for chicken population; FAOSTAT for animals slaughtered and meat harvested |
|  | 2021 | 1295516.8 | 12256.0 | 25368.0 | 2.1 | 4.4 | 53974.5 | Estimated from FAOSTAT for animals slaughtered |
| Goats | 2015 | 122598.3 | 603.0 | 7.6 | 12.5 | 26.7 | 4598.4 | Fiji agricultural census 2009 and 2020; Fiji Meat Industry report 2017 |
|  | 2016 | 126581.9 | 486.0 | 7.1 | 14.6 | 31.0 | 4749.7 | Fiji agricultural census 2009 and 2020; Fiji Meat Industry report 2017 |
|  | 2017 | 130694.8 | 801.0 | 8.7 | 10.8 | 23.0 | 4899.5 | Fiji agricultural census 2009 and 2020; Fiji Meat Industry report 2017 |
|  | 2018 | 134941.4 | 827.0 | 9.0 | 10.8 | 23.0 | 5058.7 | Fiji agricultural census 2009 and 2020; Fiji Meat Industry report 2017 |
|  | 2019 | 139326.0 | 853.9 | 9.2 | 10.8 | 23.0 | 5223.0 | Fiji agricultural census 2009 and 2020; Fiji Meat Industry report 2017 |
|  | 2020 | 143853.0 | 881.6 | 9.5 | 10.8 | 23.0 | 5392.8 | Fiji agricultural census 2009 and 2020; Fiji Meat Industry report 2017 |
|  | 2021 | 148527.1 | 910.3 | 9.9 | 10.8 | 23.0 | 5568.0 | Fiji agricultural census 2009 and 2020; Fiji Meat Industry report 2017 |
| Pigs | 2015 | 64926.6 | 16181.0 | 977.0 | 60.4 | 77.4 | 2655.0 | Fiji agricultural census 2009 and 2020; Fiji Meat Industry report 2017 |
|  | 2016 | 63569.7 | 14473.0 | 952.0 | 65.8 | 84.3 | 2593.6 | Fiji agricultural census 2009 and 2020; Fiji Meat Industry report 2017 |
|  | 2017 | 62241.2 | 14083.0 | 917.0 | 65.1 | 83.5 | 2520.1 | Fiji agricultural census 2009 and 2020; Fiji Meat Industry report 2017 |
|  | 2018 | 60940.5 | 13788.7 | 897.8 | 65.1 | 83.5 | 2467.4 | Fiji agricultural census 2009 and 2020; Fiji Meat Industry report 2017 |
|  | 2019 | 59666.9 | 13500.5 | 879.1 | 65.1 | 83.5 | 2415.8 | Fiji agricultural census 2009 and 2020; Fiji Meat Industry report 2017 |
|  | 2020 | 58420.0 | 13218.4 | 860.7 | 65.1 | 83.5 | 2365.3 | Fiji agricultural census 2009 and 2020; Fiji Meat Industry report 2017 |
|  | 2021 | 57199.1 | 12942.1 | 842.7 | 65.1 | 83.5 | 2315.9 | Fiji agricultural census 2009 and 2020; Fiji Meat Industry report 2017 |
| Sheep | 2015 | 23992.5 | 33.0 | 0.4 | 11.8 | 25.1 | 899.7 | Fiji agricultural census 2009 and 2020; Fiji Meat Industry report 2017 |
|  | 2016 | 26225.0 | 47.0 | 1.0 | 21.3 | 45.3 | 984.4 | Fiji agricultural census 2009 and 2020; Fiji Meat Industry report 2017 |
|  | 2017 | 28665.3 | 84.0 | 1.2 | 14.6 | 31.2 | 1075.5 | Fiji agricultural census 2009 and 2020; Fiji Meat Industry report 2017 |
|  | 2018 | 31332.6 | 91.8 | 1.3 | 14.6 | 31.2 | 1175.5 | Fiji agricultural census 2009 and 2020; Fiji Meat Industry report 2017 |
|  | 2019 | 34248.2 | 100.4 | 1.5 | 14.6 | 31.2 | 1284.9 | Fiji agricultural census 2009 and 2020; Fiji Meat Industry report 2017 |
|  | 2020 | 37435.0 | 109.7 | 1.6 | 14.6 | 31.2 | 1404.5 | Fiji agricultural census 2009 and 2020; Fiji Meat Industry report 2017 |
|  | 2021 | 40918.4 | 119.9 | 1.8 | 14.6 | 31.2 | 1535.2 | Fiji agricultural census 2009 and 2020; Fiji Meat Industry report 2017 |

**References**

Eurostat. (2009). Manual for the Compilation of Supply Balance Sheets for Meat.(2009). https://circabc.europa.eu/sd/a/90447c6f-5b7c-4b6f-87e9-27c5a7a5c923/ASA-TE-F-55%2520SBS%2520Manual%2520-%2520meat.doc

Fiji meat industry board. Annual Report. 2016. <https://www.parliament.gov.fj/wp-content/uploads/2021/12/181-Fiji-Meat-Industry-Board-Annual-Report-2016-1.pdf>

Food and Agriculture Organization of the United Nations. Livestock Primary in Fiji: Producing animals/slaughtered. Available online: http://www.fao.org/faostat/en/#data/QL (Accessed on 15^th^ August 2022).

Galal, S. (2005). Biodiversity in goats. Small Rumin. Res. 60(1-2), 75-81. <https://doi.org/10.1016/j.smallrumres.2005.06.021>.

Góchez, D., Raicek, M., Pinto Ferreira, J., Jeannin, M., Moulin, G., Erlacher-Vindel, E. (2019). OIE Annual Report on Antimicrobial Agents Intended for Use in Animals: Methods Used. Front. Vet. Sci. 6. doi: <https://doi.org/10.3389/fvets.2019.00317>

Ministry of Agriculture. 2020 Fiji Agriculture Census,. Volume 1: General Table & Descriptive Analysis Report.

<https://www.agriculture.gov.fj/documents/census/VOLUMEI_DESCRIPTIVEANALYSISANDGENERALTABLEREPORT.pdf> (Accessed 18th August 2022).

Ministry of Agriculture. (2009). Fiji National Agriculture Census 2009.

<http://www.fao.org/fileadmin/templates/ess/ess_test_folder/World_Census_Agriculture/Country_info_2010/Reports/Reports_3/FJI_ENG_REP_2009.pdf> (Accessed 18th August 2022).

Ting, S., Pereira, A., Alves, A.d.J., Fernandes, S., Soares, C.d.C., Soares, F.J., Henrique, O.d.C., Davis, S., Yan, J., Francis, J.R., Barnes, T.S., Jong, J.B.d.C. (2021).Antimicrobial Use in Animals in Timor-Leste Based on Veterinary Antimicrobial Imports between 2016 and 2019. Antibiotics 10(4):426. [https://doi.org/10.3390/antibiotics10040426](https://linkprotect.cudasvc.com/url?a=https%3a%2f%2fdoi.org%2f10.3390%2fantibiotics10040426&c=E,1,7OMPRbNDBBfG7u4itWAJIJu23O-272EEizvFGFfyL9cQrMvSQxmabitCgCSRli6v2C-6bJDoig8jE65vmP7fUZV5hqLnhf3NvD6uq3Igero1C0Xd6hkLvz7k&typo=1&ancr_add=1)
